# The Reindeer’s Secret: Salazinic Acid from Lichens as a Candidate Modulator of Autophagy

**DOI:** 10.3390/ijms27198621

**Published:** 2026-09-26

**Authors:** Anastasiia K. Veretina, Vera A. Alferova, Ekaterina A. Guseva, Anton R. Izzi, Arina A. Nikandrova, Gulnara K. Kudryakova, Mikhail Y. Zhitlov, Julia A. Golubeva, Valeriya B. Vays, Irina M. Vangeli, Yulia M. Khabibulina, Lora E. Bakeeva, Anna A. Lukianova, Lyamzaev G. Konstantin, Dmitry S. Karpov, Dmitrii A. Lukianov, Olga A. Dontsova, Petr V. Sergiev

**Affiliations:** 1 Center for Bio- and Medical Technologies, 121205 Moscow, Russiaarina.nikandrova@skoltech.ru (A.A.N.);; 2 Belozersky Institute of Physico-Chemical Biology, Lomonosov Moscow State University, 119991 Moscow, Russia; alferovava@gmail.com (V.A.A.); eguseva98@mail.ru (E.A.G.); lyamzaev@gmail.com (L.G.K.); 3 Department of Chemistry, Lomonosov Moscow State University, 119991 Moscow, Russia; 4 Shemyakin-Ovchinnikov Institute of Bioorganic Chemistry, Russian Academy of Science, 117997 Moscow, Russia; 5 Faculty of Bioengineering and Bioinformatics, Lomonosov Moscow State University, 119991 Moscow, Russia; 6 Engelhardt Institute of Molecular Biology, Russian Academy of Sciences, 119991 Moscow, Russia

**Keywords:** lichen extracts, novel resource of autophagy inducers, lichens, salazinic acid, *Usnea* sp.

## Abstract

Autophagy is a conserved lysosome-dependent process essential for cellular homeostasis, and pharmacological activation of autophagy has emerged as a promising therapeutic strategy for age-related, neurodegenerative, metabolic, and malignant diseases. Despite the growing interest in natural autophagy modulators, lichens remain largely unexplored as a source of autophagy-inducing compounds. Here, we tested ethanolic extracts from four commercially available lichens using LC3-II accumulation as a primary readout. *Usnea* sp. was identified as the most active species. Activity-guided fractionation followed by HRMS and NMR analyses identified salazinic acid as the active metabolite. Salazinic acid increased LC3-II accumulation, enhanced autophagic flux in the presence of bafilomycin A1, and promoted autophagosome formation as confirmed by transmission electron microscopy. The compound exhibited low cytotoxicity in human cell lines at concentrations exceeding those required for autophagy induction. Mechanistically, salazinic acid activated the phosphorylation of AMPK, ULK1, and AKT; however, it retained its ability to stimulate autophagic flux in AMPK- and ULK1-deficient cells, indicating that its activity is not strictly dependent on the canonical AMPK–ULK1 signaling pathway. Collectively, our findings identify salazinic acid as a previously unrecognized inducer of productive autophagic flux and demonstrate that lichen secondary metabolites represent a promising and largely untapped source of novel autophagy-modulating compounds.

## 1. Introduction

Autophagy is a highly conserved lysosome-dependent pathway that enables eukaryotic cells to degrade damaged organelles, protein aggregates, and other cytoplasmic components [1]. Beyond its housekeeping function, autophagy plays a central role in cellular adaptation to nutrient deprivation, oxidative stress, hypoxia, mitochondrial dysfunction, and other stress conditions [2,3,4,5]. Impaired autophagy contributes to the pathogenesis of numerous disorders, including neurodegenerative, metabolic, and malignant diseases, as well as aging [6,7,8], making pharmacological activation of autophagy an attractive therapeutic strategy.

A broad range of natural [9] and synthetic [10] autophagy activators has been identified. Synthetic compounds, including mTOR inhibitors and direct autophagy activators, can induce strong autophagic responses; however, their clinical application may be limited by toxicity, poor specificity, or undesirable effects associated with prolonged pathway activation [11,12]. In contrast, naturally occurring compounds often exhibit favorable pharmacological profiles and have attracted considerable interest as potential autophagy-modulating agents [13]. Several natural molecules, including urolithin A [13], spermidine [14], and resveratrol [15], have demonstrated beneficial effects associated with autophagy regulation. Nevertheless, the limited potency and bioavailability [16] of many natural compounds highlight the need to identify new autophagy modulators with improved efficacy and therapeutic potential.

Given that autophagy is a fundamental, evolutionarily conserved mechanism that enables cellular adaptation to environmental stress, organisms that have evolved under chronic stress conditions may represent particularly promising sources of autophagy-modulating compounds. Lichens are highly stress-tolerant terrestrial organisms, capable of surviving repeated cycles of desiccation and rehydration, prolonged nutrient limitation, intense ultraviolet radiation, and extreme temperature fluctuations while maintaining long-term viability [17,18,19]. Moreover, their symbiotic nature, combining partners with fundamentally different metabolic capabilities, gives rise to a unique repertoire of secondary metabolites [20].

More than 1000 lichen secondary metabolites have been identified to date, with phenolic compounds representing one of the most prominent groups, including structurally distinctive classes such as depsides, depsidones, dibenzofurans, xanthones, and pulvinic acid derivatives [21,22,23,24]. Although these metabolites have been extensively investigated for their antimicrobial, antioxidant, anti-inflammatory, antiviral, and antitumor properties [25,26], their ability to regulate autophagy has received limited attention, with usnic acid being the most noticed compound [27]. At the same time, a number of naturally occurring autophagy modulators, including the gut microbiota-derived metabolite urolithin A [13], belong to the broader class of phenolic compounds.

In this study, we used a bioactivity-guided screening strategy [28] to identify autophagy-inducing metabolites from lichen extracts. Salazinic acid was detected as the main active compound in *Usnea* sp. extract. The effects of salazinic acid on autophagic flux, cellular ultrastructure, cytotoxicity, and autophagy-related signaling pathways were comprehensively evaluated. Notably, testing a relatively small collection of lichen extracts was sufficient to identify a potent autophagy-inducing metabolite. Our findings demonstrate that lichen secondary metabolites constitute a promising and largely untapped source of structurally diverse autophagy modulators and provide a framework for the discovery of bioactive natural products from lichens.

## 2. Results

### 2.1. Collection and Identification of Lichens

**Figure 1:**
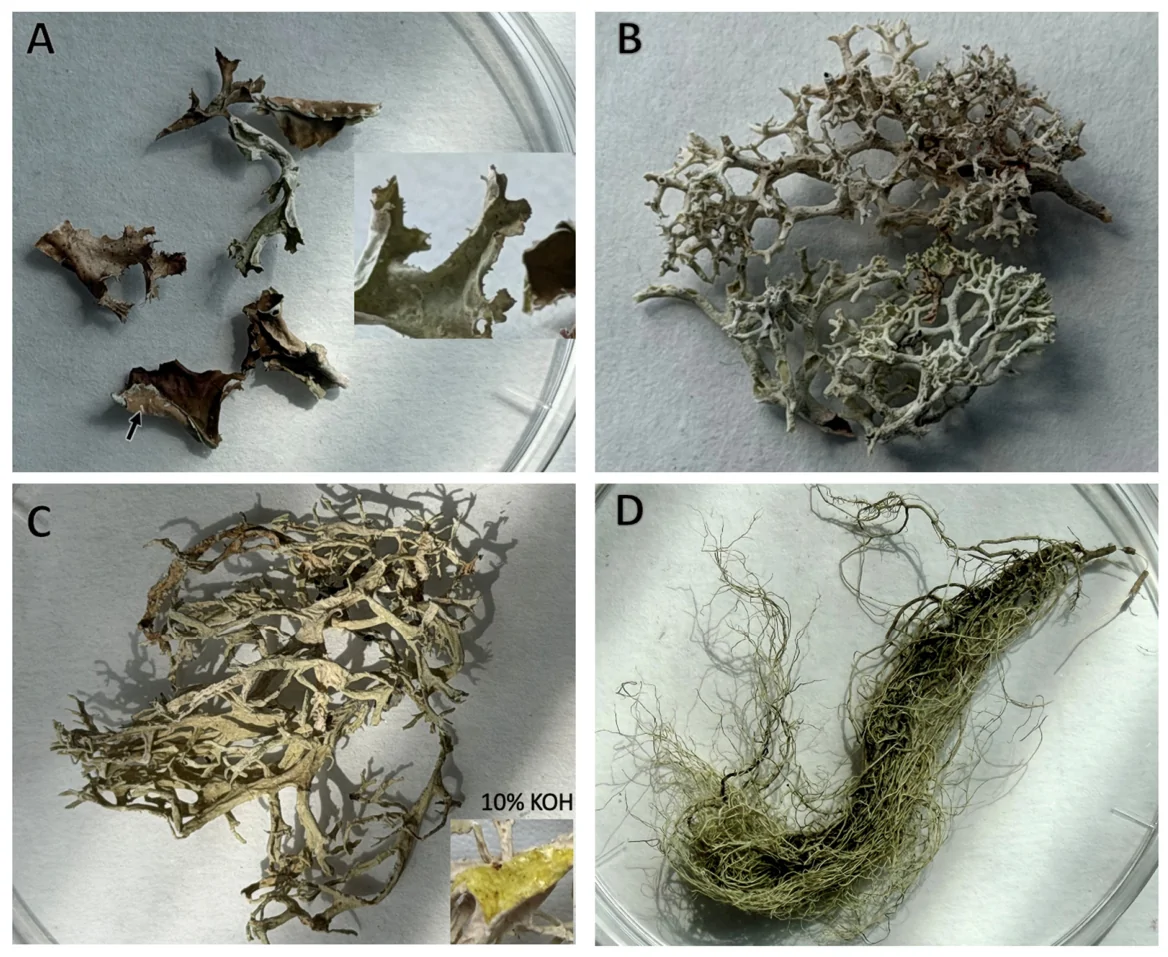
Macro-morphological features used for the identification of the commercial lichen raw materials: (**A**) *Cetraria islandica*: rounded-angular to cylindrical thallus with numerous spinules and cilia, olive-brown above, pale greyish-whitish below with distinct white spots (cyphellae/pseudocyphellae, indicated by an arrow) [29,30]. (**B**) *Cladonia stellaris*: fruticose, whitish-grey-yellowish thallus with densely and repeatedly divided branches; apical branchlets short, thick, rounded and characteristically star-like; no apothecia observed in the material [29,31]. (**C**) *Evernia prunastri*: fruticulose-ascending, flat-lobed thallus with upper surface differing in color from the lower, lobes gathered into an erect bushy tuft without cilia at the margins; lower surface with a cortical layer which turns yellow with 10% KOH (see magnified inset); upper surface greyish-green, lower lighter; thallus without apothecia, with soredia and isidia, lobes flat, ribbon-like, dorsiventral [29,30]. (**D**) *Usnea* sp.: fruticose-pendent, round-lobed thallus with a dense axial cord in the central medulla; surface smooth, greyish-green; thallus elongated, composed of elongated greyish-green hair-like branches [29,30].

Four distinct types of commercial lichen raw materials were purchased from the “Russkie Korni” (Russian Roots) specialized herbal retail chain (Moscow, Russia). Identification was performed on a morphological basis, utilizing distinctive macro-morphological features of the thalli. The studied materials were taxonomically determined as *Cetraria islandica*, *Cladonia stellaris*, *Evernia* sp., and *Usnea* sp. Photographic documentation of the raw materials is provided in Figure 1.

### 2.2. Primary Screening of Lichen Extracts for Their Ability to Induce Autophagy

To screen for autophagy activators from lichens, we adapted our previously described protocol used for medicinal plants [28]. Ethanol extraction was selected as a rapid and scalable approach for obtaining concentrated extracts enriched in lichen secondary metabolites. Dried lichen material (1 g) was extracted by boiling in 30 mL of 90% ethanol, and the resulting extracts were tested for their ability to modulate autophagy in SH-SY5Y cells. SH-SY5Y cells are a widely used human neuroblastoma-derived neuronal model for studying neuronal biology, cellular stress responses, and neurodegeneration-related mechanisms. Pharmacological activation of autophagy has been proposed as a potential therapeutic strategy for neurodegenerative diseases, and autophagy-inducing compounds have been investigated in SH-SY5Y cells in models of Parkinson’s disease and neurotoxic stress [32,33]. Therefore, SH-SY5Y cells provide a relevant and well-established model for the initial characterization of novel autophagy-modulating compounds.

Autophagy-related changes were initially assessed by monitoring LC3-I to LC3-II conversion using immunoblotting analysis. Lipidation of LC3-I to form LC3-II accompanies its incorporation into the membrane of the growing phagophore and is widely used as a molecular marker associated with autophagosome formation [34]. For this primary comparative screening, the LC3-II/LC3-I ratio was used to rank lichen extracts tested in parallel under identical experimental conditions. Although normalization of LC3-II to a loading control is preferable for quantitative assessment of LC3-II abundance, changes in both LC3-I and LC3-II can provide informative complementary information on LC3 processing [35]. The LC3-II/LC3-I ratio in this experiment was therefore used only as a screening parameter and not as a measure of autophagic flux. To validate the applicability of the extraction and screening procedure to lichen material, *Cladonia stellaris* was selected as a model species. This lichen is widespread throughout boreal and Arctic regions and is known to contain a variety of biologically active secondary metabolites, including usnic acid [36]. Previous studies have shown that usnic acid exhibits antitumor, antioxidant, and anti-inflammatory activities, effects that have been partially attributed to its ability to induce autophagy [37].

**Figure 2:**
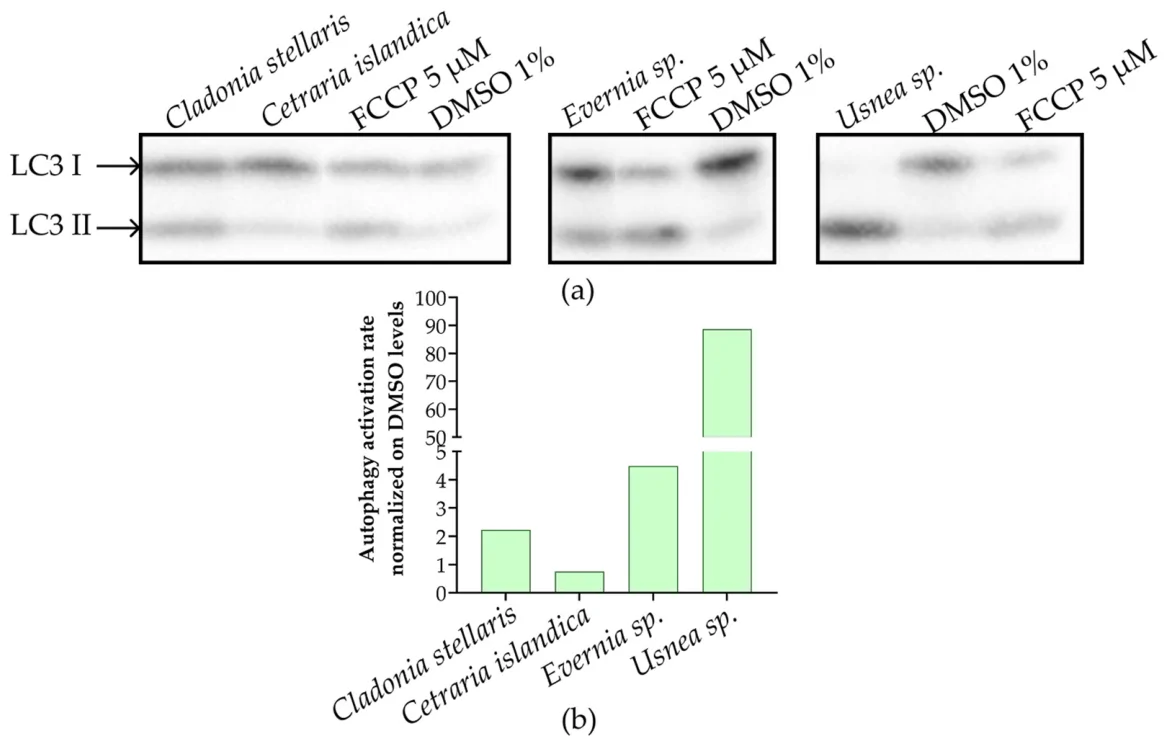
Primary screening of the ability of ethanolic extracts from four lichen species to modulate autophagy, assessed by immunoblotting analysis of LC3-I/II. (**a**) Immunoblotting analysis of the autophagy-inducing abilities of ethanolic extracts from various lichens (full images are shown in Figure S1). (**b**) Bar graph indicating the LC3 II/I ratio in cells after treatment with lichen extracts, normalized to the corresponding ratio in negative controls (DMSO 1%). The ratio was used as a comparative screening parameter for extracts tested in parallel and was not used as a measure of autophagic flux.

Treatment of SH-SY5Y cells with the ethanolic extract of *C. stellaris* resulted in an increase in LC3-II levels compared with untreated controls, indicating that the proposed extraction and screening workflow is suitable for detecting autophagy-modulating activity in lichen-derived samples (Figure 2a and Figure S1).

Following method validation, three additional lichen species were screened using the same experimental approach (Figure 2a; the exact values of relative protein abundance obtained from the analysis of all images are provided in Table S3 and Figure S1). Initial experiments were performed using 5 μL of extract per well. Among the tested species, only *Usnea* sp. produced the largest increase in the LC3-II/LC3-I ratio relative to the negative control (Figure 2b) and was therefore selected for subsequent activity-guided fractionation and detailed characterization.

### 2.3. Identification of the Active Compound in Usnea sp.

**Figure 3:**
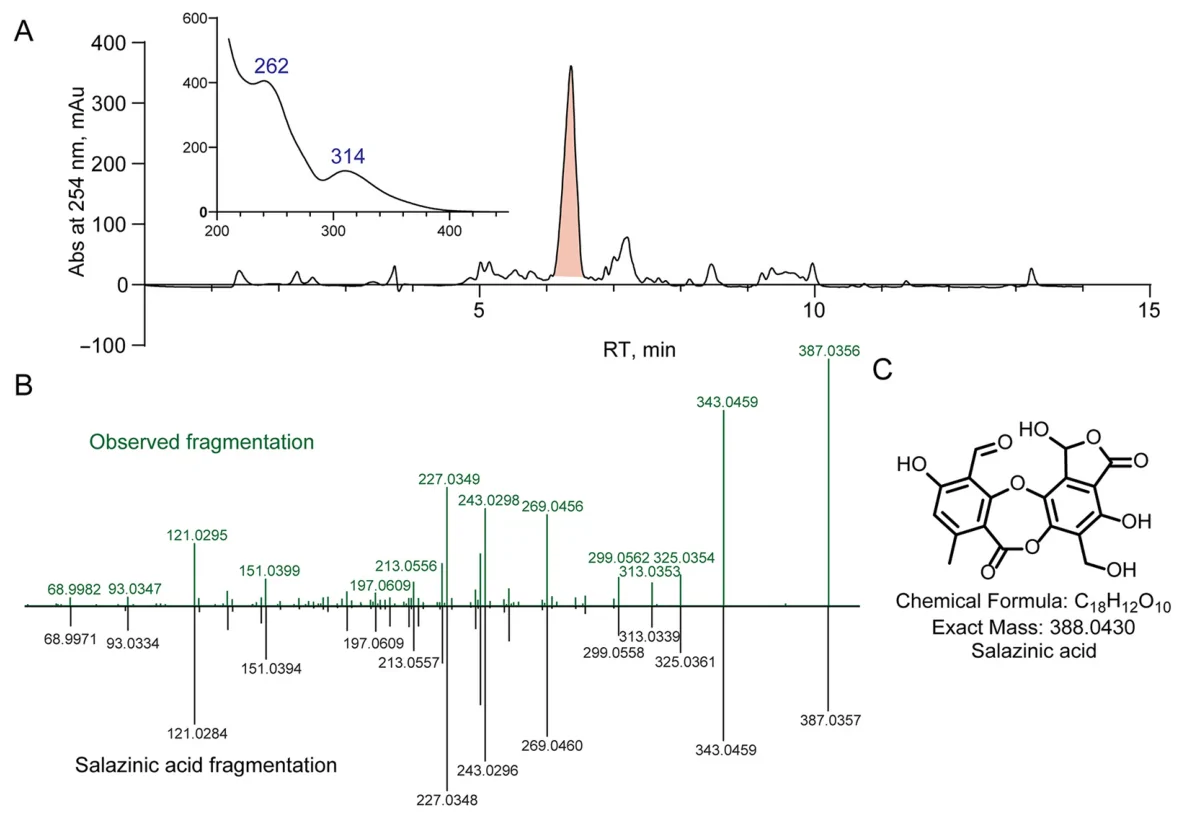
Structure elucidation of salazinic acid. (**A**) HPLC profile of *Usnea* sp. ethanolic extract; the active peak is highlighted in orange. (**B**) MS/MS comparison of the observed ion in negative-ion mode (*m*/*z* 387.0353) with the deposited GNPS reference spectrum for salazinic acid [M-H]^−^ (GNPS-LIBRARY:accession: CCMSLIB00005719564, *m*/*z* 387.0350). (**C**) Chemical structure of salazinic acid.

The ethanolic extract of *Usnea* sp. was subjected to activity-guided fractionation by HPLC with UV detection. The active component displayed UV–Vis absorption maxima at 262 and 314 nm (Figure 3A). LC–HRMS analysis of the isolated compound in negative-ion mode revealed a [M-H]^−^ ion at *m*/*z* 387.0353, while no corresponding positive-mode adducts were detected. The measured mass is consistent with the molecular formula C_18_H_12_O_10_ (calculated [M-H]^−^ 387.0352, Δ 0.3 ppm). The MS/MS fragmentation pattern (Figure 3B and Figure S2) matched that of deprotonated salazinic acid (Figure 3C). The identity of the isolated compound as salazinic acid was further corroborated by comparison of its NMR data with published values [38] (Tables S1 and S2, Figure S5).

### 2.4. Salazinic Acid Extracted from Usnea sp. Increases Autophagic Flux

**Figure 4:**
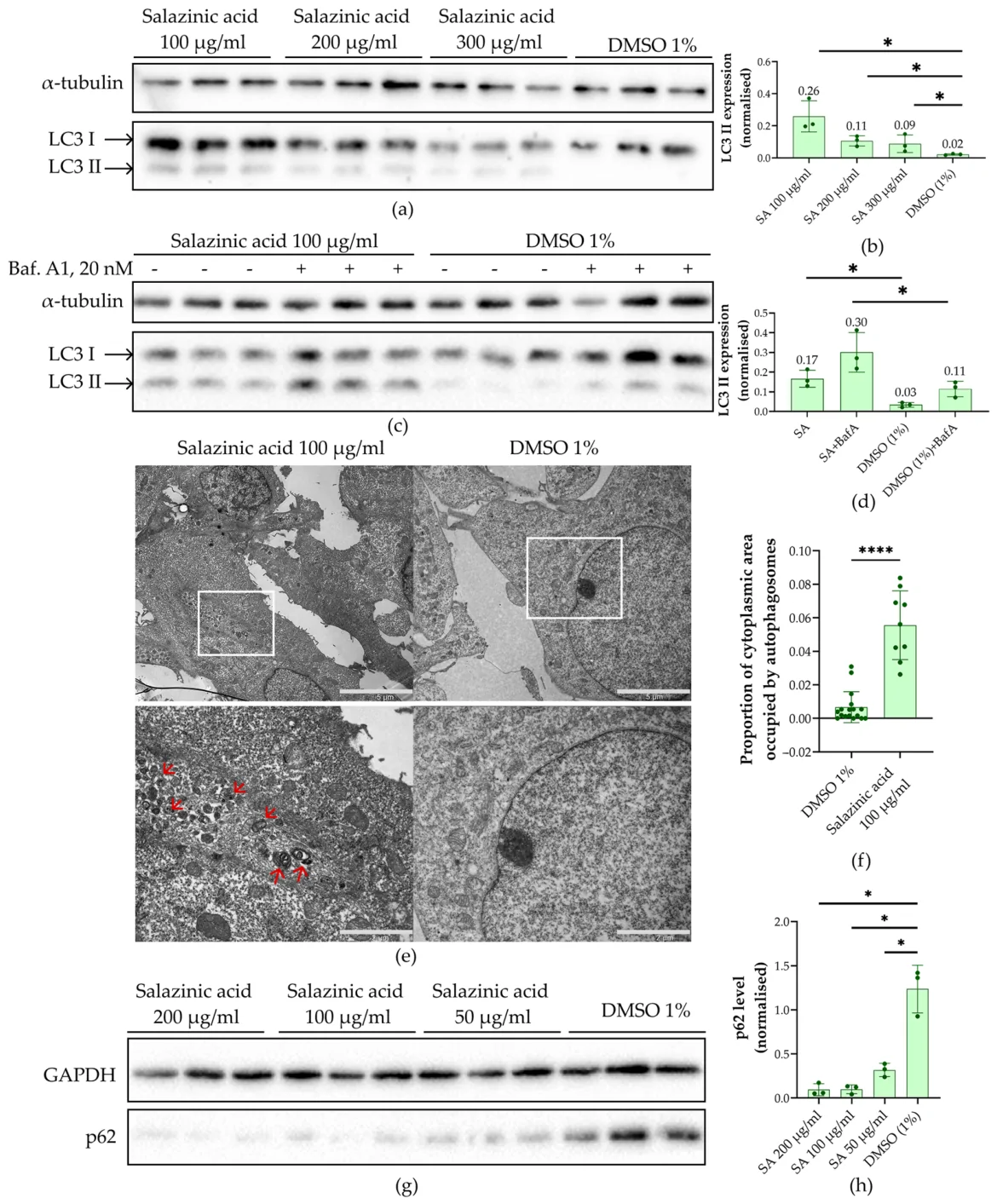
Effects of salazinic acid on autophagic flux in SH-SY5Y cells and autophagosome abundance in N2a cells. Panels (**a**–**d**,**g**,**h**) show experiments performed in SH-SY5Y cells, whereas panels (**e**,**f**) show transmission electron microscopy analysis performed in N2a cells. (**a**) Immunoblotting analysis of autophagy activity in cells after treatment with salazinic acid at different concentrations or 1% DMSO for 16 h. (**b**) Bar graph indicating levels of LC3 II normalized to α-tubulin in cells after treatment with different concentrations of salazinic acid or 1% DMSO for 16 h. (**c**) Immunoblotting analysis of autophagic flux in cells after treatment with salazinic acid (100 µg/mL) or DMSO 1% for 16 h in the presence or absence of Bafilomycin A1 (2 h, Baf. A1). (**d**) Bar graph indicating levels of LC3 II normalized to α-tubulin in cells after treatment with salazinic acid (100 µg/mL) or DMSO 1% for 16 h in the presence or absence of Bafilomycin A1 (2 h, BafA). SA—salazinic acid. (**e**) Electron microphotographs showing accumulation of autophagosomes after treatment with salazinic acid. Grey bars indicate 5 μm (upper panel) and 2 μm (bottom panel). Red arrows indicate autophagosomes. White frames indicate magnified areas (bottom panel). (**f**) Bar graph representing proportion of cytoplasmic area occupied by autophagosomes in cells after salazinic acid treatment (*n* = 10). (**g**) Immunoblotting analysis of p62 degradation in cells after treatment with salazinic acid or DMSO 1% for 16 h. (**h**) Bar graph indicating levels of p62 normalized to GAPDH in cells after treatment with salazinic acid or DMSO 1% for 16 h. The results of a one-sided Mann–Whitney U test are indicated on the graph. Here and further: * represents *p*-value = 0.05, ****—*p*-value < 0.0001; each dot on the blot represents an independent biological replicate. SA—salazinic acid.

Following the isolation and purification of salazinic acid from *Usnea* sp., we investigated its potential as an autophagy activator. We tested a wide range of salazinic acid concentrations for their ability to induce LC3-II accumulation (Figure S3), selecting the 100–300 μg/mL range for further experiments, as higher concentrations caused changes in cell morphology. Since it was important for us to ensure the reproducibility of the active component isolation from *Usnea* sp., at this stage, we re-purified salazinic acid and then evaluated its ability to induce autophagy over a range of incubation times (8 or 16 h). Our results demonstrate that, unlike rapidly acting autophagy inducers such as Torin1 [39], salazinic acid did not induce an immediate response. Instead, a significant increase in LC3-II accumulation was observed only after at least 16 h of treatment (Figure 4a,b; the exact values of relative protein abundance obtained from the analysis of all images, as well as the U-statistic values, are provided in Table S3), while 8 h incubation was not enough to detect significant autophagy activation (Figure S4a,b). Notably, this effect was detected throughout the entire concentration range examined (100–300 μg/mL) (Figure 4a,b).

To verify that salazinic acid stimulates autophagic activity rather than inhibiting the late stages of autophagosome degradation, we assessed its effects in the presence of bafilomycin A1, a well-established specific inhibitor of vacuolar H+-ATPase (V-ATPase) that prevents lysosomal acidification, thereby inhibiting lysosomal degradation and blocking autophagic flux [40]. As expected, treatment with salazinic acid resulted in a statistically significant increase in LC3-II levels compared with the corresponding control under all experimental conditions. Importantly, co-treatment with salazinic acid and bafilomycin A1 produced a significantly greater accumulation of LC3-II than treatment with salazinic acid alone (Figure 4c,d). This additive effect indicates that salazinic acid enhances autophagic flux rather than impairing autophagosome degradation, confirming its role as an autophagy activator. Consistent with this interpretation, salazinic acid treatment significantly decreased p62 levels at all tested concentrations (Figure 4g,h), indicating enhanced autophagic degradation. This decrease in p62 also suggests that the lower LC3-II levels observed at higher concentrations of salazinic acid are unlikely to result from impaired lysosomal degradation and are instead consistent with increased autophagic turnover.

To further demonstrate that the observed effects are not restricted to a specific cell line or species, we confirmed that salazinic acid increased autophagic flux in mouse neuroblastoma N2a cells (Figure S4c,d). As an independent approach to characterize the cellular response to salazinic acid, we next performed transmission electron microscopy in N2a cells, followed by quantitative analysis of autophagosome abundance (Figure 4e,f). Ultrastructural examination revealed numerous double-membrane vesicles with diameters ranging from approximately 70 to 300 nm that contained folded, electron-dense cytoplasmic material, consistent with the characteristic morphology of autophagosomes. Quantitative analysis demonstrated a statistically significant increase in the number of these double-membrane vesicles in the cytoplasm of salazinic acid-treated cells compared with untreated controls.

Collectively, the biochemical experiments in SH-SY5Y cells support increased autophagic flux, while the complementary ultrastructural analysis in N2a cells demonstrates increased abundance of structures with autophagosome-like morphology following salazinic acid treatment.

### 2.5. Cytotoxicity of Salazinic Acid

**Figure 5:**
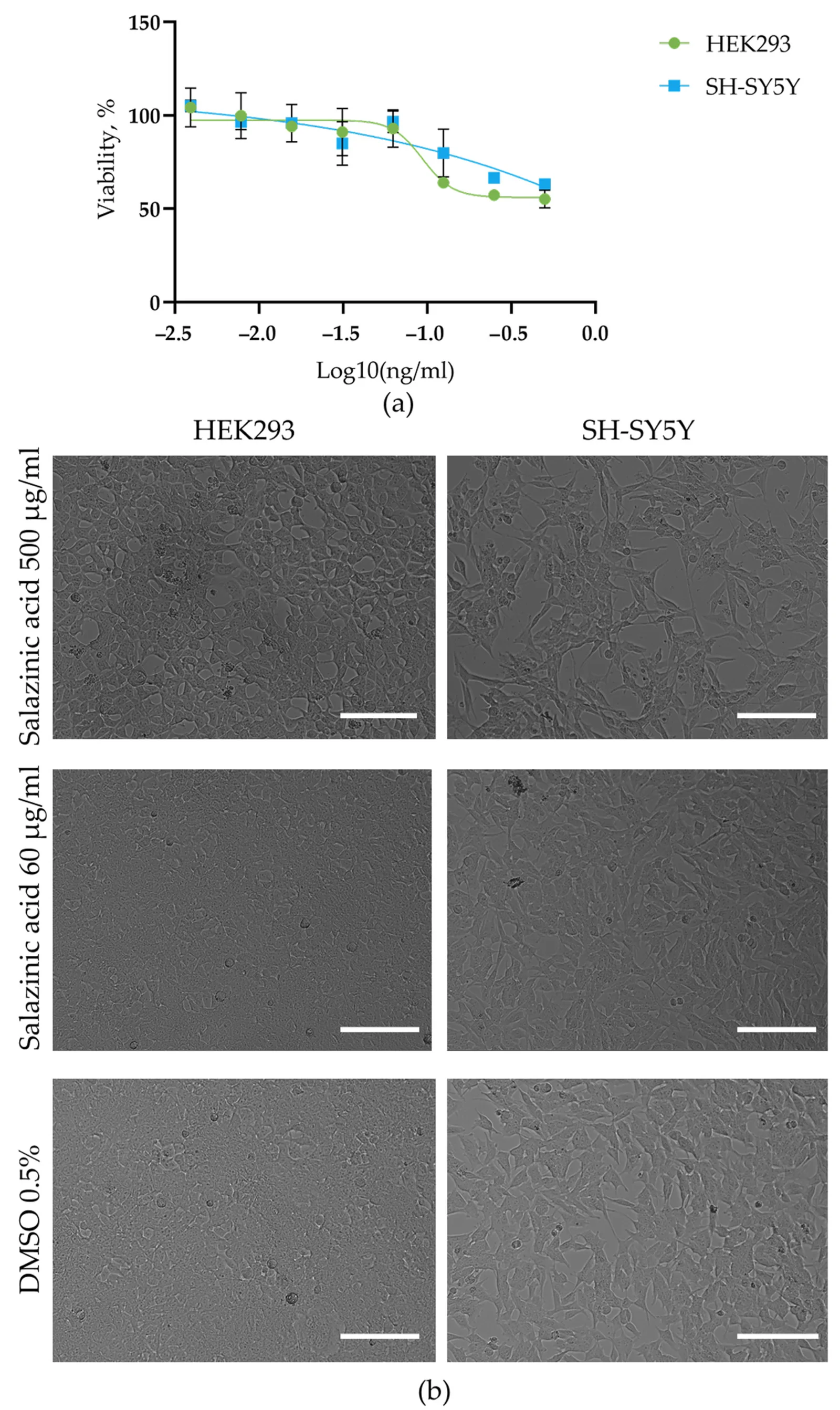
Salazinic acid exhibits low cytotoxicity. (**a**) Viability of human neuroblastoma (SH-SY5Y) and embryonic kidney (HEK293) cells incubated with salazinic acid for 24 h. (**b**) Microphotographs of SH-SY5Y and HEK293 cells incubated with salazinic acid and DMSO. White bars indicate 100 μm. Error bars indicate SD.

Cytotoxicity testing on human neuroblastoma and human embryonic kidney cell lines demonstrated that salazinic acid exhibited low cytotoxicity. Within the tested concentration range (3.9–500 µg/mL), inhibition of cell growth and a reduction in cell number were observed only at the highest concentrations (125–500 µg/mL) (Figure 5). However, the cells retained normal morphology, and no morphological signs of apoptosis were detected. At lower concentrations, treatment with salazinic acid did not result in any significant changes in cell number, morphology, or metabolic activity.

Overall, salazinic acid did not exhibit detectable cytotoxic effects on human cells at concentrations up to fivefold higher than the minimum concentration required to activate autophagy.

### 2.6. Cellular Signaling Associated with Salazinic Acid Treatment

We further investigated the mechanism underlying salazinic acid-induced autophagy by analyzing the phosphorylation status of key components of the signaling pathways regulating autophagy in cultured cells.

**Figure 6:**
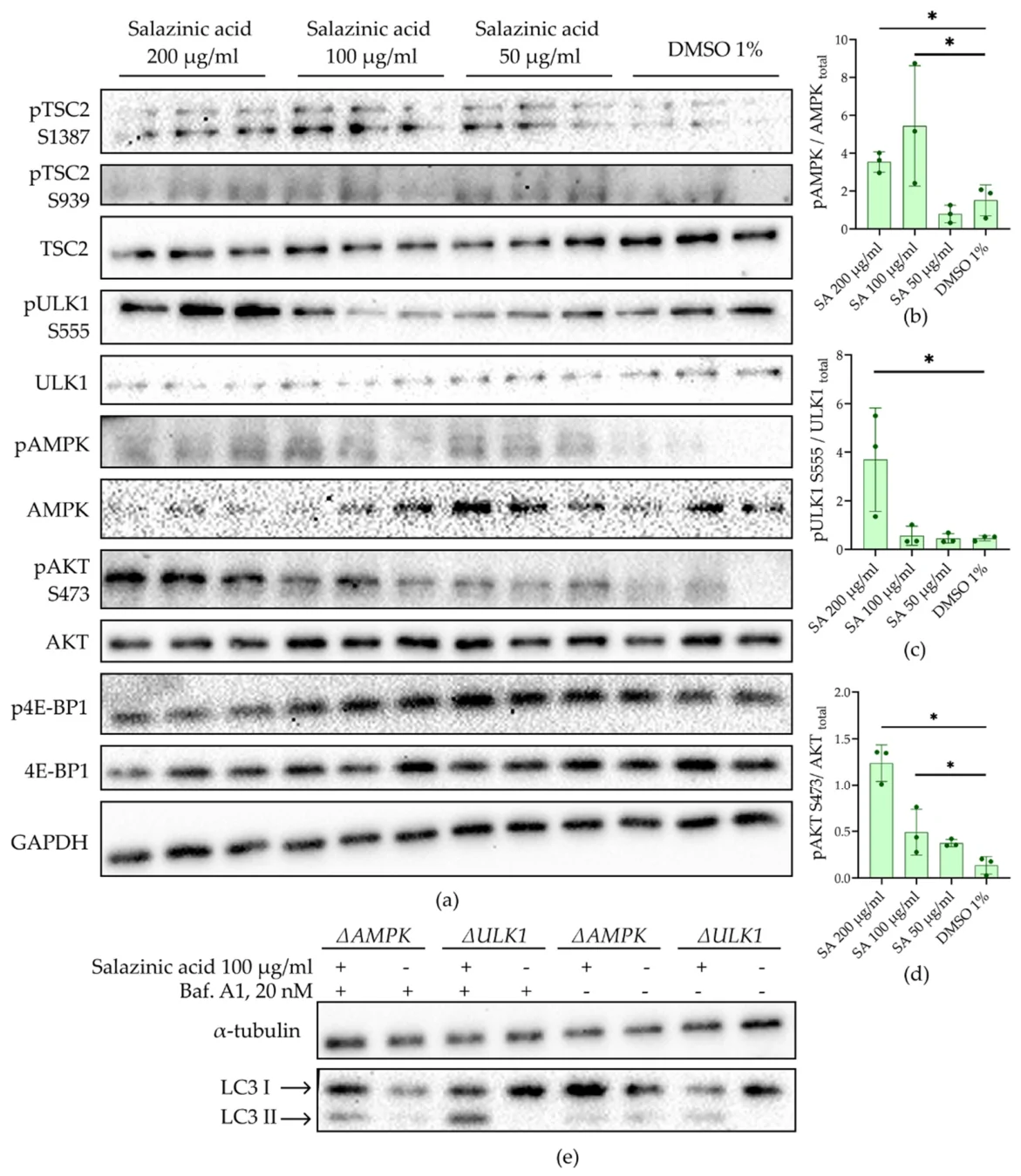
Salazinic acid activates autophagy and is associated with AMPK–ULK1 signaling. (**a**) Immunoblotting analysis for various protein regulators of the autophagy cascade. (**b**–**d**). Changes in phosphorylation status of AMPK (**b**), ULK1 (**c**), and AKT (**d**). Data represent mean ± SD (n = 3). The results of Mann–Whitney tests are indicated on the graph: *—*p*-value = 0.05. (**e**) Immunoblotting analysis of the key marker of autophagy (LC3) in AMPK-knockout and ULK1-knockout SH-SY5Y cells after incubation with salazinic acid in the presence or absence of bafilomycin A1. The results of a one-sided Mann–Whitney U test are indicated on the graph. SA—salazinic acid.

Treatment with salazinic acid resulted in a significant increase in the activating phosphorylation of AMPK, indicating enhanced AMPK activity in cells (Figure 6a,b). This conclusion is further supported by the increased phosphorylation of ULK1 at Ser555 (Figure 6a,c), a well-established AMPK-dependent phosphorylation site that promotes ULK1 activation and autophagy initiation [41]. TSC2 is another downstream target of AMPK and can be phosphorylated by AMPK at Ser1387, promoting TSC2 activity and subsequent inhibition of Rheb and mTORC1 signaling [42]. We observed a trend toward increased phosphorylation of TSC2 at Ser1387, accompanied by a decreasing trend in phosphorylation of 4E-BP1, a downstream target of mTORC1 (Figure 6a,d). These findings are consistent with reduced mTORC1 activity and further support the involvement of AMPK-associated signaling in the autophagy response to salazinic acid.

In addition to activating the AMPK–ULK1 signaling axis, salazinic acid also significantly increased the phosphorylation of AKT at Ser473 (Figure 6a,d). Phosphorylation of this residue is mediated by mTORC2 [43] and is required for full activation of AKT. Activated AKT functions as an upstream positive regulator of the inhibitory AKT/mTOR signaling pathway, which suppresses autophagy. The increase in AKT Ser473 phosphorylation was not accompanied by evidence of increased mTORC1 activity, as phosphorylation of 4E-BP1 showed a tendency to decrease. Thus, AKT activation appears to represent an associated signaling event whose functional relationship to salazinic acid-induced autophagy remains unresolved.

To investigate the contribution of the AMPK–ULK1 signaling pathway to the autophagy-inducing activity of salazinic acid, we examined its effects in *ΔAMPK* and *ΔULK1* knockout cell lines (Figure 6e). Autophagic flux was assessed by monitoring LC3-II accumulation in the presence and absence of the lysosomal inhibitor bafilomycin A1. Salazinic acid retained its ability to stimulate autophagic flux in both knockout cell lines, as evidenced by the increased accumulation of LC3-II. These findings indicate that the loss of either AMPK or ULK1 individually is insufficient to abolish the autophagic response to salazinic acid. To further assess the response under combined inhibition of the AMPK–ULK1 axis, we treated *ΔAMPK* cells with the ULK1 inhibitor ULK101 (10 µM). Salazinic acid retained its ability to increase LC3-II levels under combined AMPK and ULK1 inhibition (Figure S7), further supporting the conclusion that inhibition of the AMPK–ULK1 pathway does not abolish the response to salazinic acid. Thus, although salazinic acid treatment is accompanied by activation of AMPK–ULK1 signaling, these findings do not support an essential role for this pathway in mediating its autophagy-inducing activity.

## 3. Discussion

Despite the remarkable diversity of lichen secondary metabolites, their ability to regulate autophagy remains largely unexplored. To date, usnic acid is the only lichen metabolite reported to modulate autophagy; however, its mechanism of action remains controversial. While early studies have described usnic acid as an autophagy inducer [37], later it was shown that it promotes autophagosome accumulation by impairing lysosomal acidification and blocking autophagic degradation [27,44], thereby inhibiting autophagic flux. In the present study, we screened crude extracts from four lichen species for their ability to induce autophagy and identified salazinic acid as a novel inducer of productive autophagic flux.

Salazinic acid belongs to the depsidones, one of the characteristic classes of lichen secondary metabolites [45]. Although several lichen depsidones, including norstictic, stictic, physodic, protocetraric, and fumarprotocetraric acids, have been investigated for their biological activities, previous studies have focused mainly on their antimicrobial, antioxidant, anti-inflammatory, antiviral, and cytotoxic properties [46]. In the present study, we demonstrate for the first time that salazinic acid, a lichen-derived depsidone, acts as an inducer of autophagic flux. Our findings raise the possibility that the ability to modulate autophagy may be shared by other members of the depsidone family, making them promising candidates for future investigation.

Previous studies have reported antioxidant [47], antimicrobial [48], and antiproliferative [47] activities of salazinic acid, similar to those described for several other depsidones. In addition, in vivo studies have demonstrated that salazinic acid exhibits relatively low toxicity (LD50 > 100 mg/kg for rats) [49]. Our findings are consistent with these observations, as no pronounced cytotoxicity was detected at concentrations that effectively induced autophagy. Importantly, in contrast to the controversial findings reported for usnic acid, our bafilomycin A1 experiments demonstrated that salazinic acid increased LC3-II accumulation by stimulating autophagosome formation rather than by inhibiting lysosomal degradation in human and mouse neuroblastoma cell lines. These results indicate activation of productive autophagic flux and further support the conclusion that salazinic acid functions as an autophagy inducer rather than an autophagy inhibitor. The increase in autophagic flux observed in both SH-SY5Y and N2a cells was further supported by ultrastructural analysis in N2a cells, which revealed an increased abundance of autophagosomes. Together, these complementary biochemical and ultrastructural data support the induction of autophagic activity by salazinic acid across two neuronal cell models.

Mechanistically, treatment with salazinic acid was accompanied by increased phosphorylation of AMPK and ULK1, consistent with activation of one of the major signaling pathways involved in autophagy initiation. At the same time, we observed increased phosphorylation of AKT at Ser473, a modification commonly regarded as a marker of mTORC2-dependent AKT activation. Although AMPK and AKT are often considered functionally opposing regulators of autophagy, simultaneous activation of these pathways has been reported under metabolic stress conditions [50]. In our experiments, increased AKT Ser473 phosphorylation was not accompanied by increased phosphorylation of the mTORC1 downstream target 4E-BP1, suggesting that the functional significance of AKT activation in the response to salazinic acid remains unclear. Notably, salazinic acid retained its ability to induce autophagy in cell lines with inactivation of either AMPK or ULK1. These findings indicate that loss of either protein individually is insufficient to abolish the autophagic response to salazinic acid. Furthermore, salazinic acid retained its activity under combined AMPK and ULK1 inhibition in *ΔAMPK* cells treated with the ULK1 inhibitor ULK101. Together, these findings indicate that inhibition of the AMPK–ULK1 pathway does not abolish the autophagy-inducing activity of salazinic acid. Thus, although activation of AMPK–ULK1 signaling accompanies the cellular response to salazinic acid, our data do not support an essential role for this pathway in mediating autophagy induction. The precise molecular mechanism underlying the autophagy-inducing activity of salazinic acid remains to be determined.

In conclusion, the present study identifies salazinic acid as a previously unrecognized inducer of productive autophagic flux and substantially expands the current understanding of its biological activity. Our findings further demonstrate that lichen secondary metabolites represent a promising yet largely unexplored source of novel autophagy modulators. These results provide a foundation for the future discovery of bioactive compounds from lichens and support their potential development as therapeutic agents targeting autophagy.

## 4. Materials and Methods

### 4.1. Identification of Lichens

The four types of commercial lichen raw materials were obtained as a single large batch from the “Russkie Korni” (Russian Roots) specialized herbal retail chain (Moscow, Russia). The supplier provided the initial species names. To confirm these designations, we performed an independent re-identification based on morphological and biochemical characters using dichotomous identification keys [29,30,31].

Identification was carried out based on morphological traits using the following identification keys [29,30,31]; a binocular microscope and 10% KOH solution were used for examination.

### 4.2. Ethanolic Extraction

The lichen material was acquired from the ‘Russkie Korni’ store (https://magazintrav.ru). The plants were ground with an ULTRA-TURRAX^®^ Tube Drive (IKA, Staufen, Germany) homogenizer to create a fine, homogeneous powder in order to extract them. Mixing 1 g of powder with 30 mL of 90% EtOH is the next step. The suspension was heated in a microwave for 5 min total without intensive boiling. The extracted sample was cooled and centrifuged to separate the supernatant from the sediment, and then the supernatant was used. Extracts were kept at +4 °C in containers that were tightly closed.

To isolate relatively large amounts of salazinic acid, dried strings of lichen species were loaded into Asahina’s apparatus [51] with a main flask volume of 4 L and extracted with ethanol. The ethanolic extract was then dried under reduced pressure. The resulting brown paste was washed with water and o-xylene (Chimmed, Moscow, Russia) and then subjected to preparative HPLC.

### 4.3. Generation and Maintenance of Mammalian Cell Lines

The human neuroblastoma (SH-SY5Y, the cell line was obtained from Karagyaur Maxim Nikolaevich, Department of Biochemistry and Regenerative Biomedicine, Faculty of Fundamental Medicine, Lomonosov Moscow State University), human embryonic kidney (HEK293, the cell line was obtained from Ekaterina Sergeevna Knyazhanskaya Faculty of Chemistry, Lomonosov Moscow State University) and mouse neuroblastoma (N2a, the cell line was obtained from Anna Vladimirovna Bacheva Faculty of Chemistry, Lomonosov Moscow State University) cell lines were cultured in DMEM/F12 medium (Gibco™: 11320033, Thermo Fisher Scientific, Waltham, MA, USA), containing 10% FBS (Gibco™: 16000044, Thermo Fisher Scientific, Waltham, MA, USA), 100 U/mL of penicillin and 100 µg/mL of streptomycin (Gibco™: 15140122, Thermo Fisher Scientific, Waltham, MA, USA), 1% glutaMAX (Gibco™: 35050061, Thermo Fisher Scientific, Waltham, MA, USA) at 37 °C, 5% CO_2_. Harvesting was performed when reaching 70–80% confluence via trypsinization.

To investigate the mechanism of action of salazinic acid, we used SH-SY5Y cell lines with individually inactivated AMPK or ULK1 genes, generated previously [52,53,54].

### 4.4. Immunoblotting

For investigation and characterisation of autophagy-activating properties, the required volume of extract, fraction as a whole, or pure compound was lyophilized using the Labconco FreeZone 2.5L dryer (Labconco, KC, USA)and then dissolved in 100 µL medium with 1% dimethyl sulfoxide (DMSO, Component-reagent, Moscow, Russia). The mixture was applied to the cells 24 h after plating. Carbonyl cyanide p-trifluoromethoxyphenylhydrazone (FCCP; APExBIO: B5004, Hong Kong, China) was used as a positive control. After 8 or 16 h, cells were harvested and analyzed. For the autophagic flux analysis, cells were incubated with salazinic acid or DMSO for 14 h, after which bafilomycin A1 (20 nM) was added to the medium containing salazinic acid or DMSO for an additional 2 h. After that, cells were harvested. 

For the pharmacological inhibition of ULK1 [55], cells were incubated with the ULK1 inhibitor ULK101 (10 µM; HY-114490, MedChem Express, Monmouth Junction, NJ, USA) for 16 h in the presence or absence of salazinic acid.

For stain-free total protein normalization, 2,2,2-trichloroethanol (Macklin, Shanghai, China) was added to the polyacrylamide gel according to the manufacturer’s recommendations.

Immunoblotting of cell lysates was performed as previously described [52]. Briefly, for immunoblot analysis, cells of SH-SY5Y were lysed in a RIPA buffer (150 mM sodium chloride, 50 mM Tris-HCl pH 8.0, 0.5% Nonidet P-40, 1% sodium deoxycholate, 0.5% SDS). Obtained cell lysates were applied to the 12% SDS-PAGE gel, followed by wet transfer to the PVDF membrane (0.45 μm, Thermo Fisher Scientific, Waltham, MA, USA). After that, the membrane was blocked for 1 h in TBST (10 mM Tris-HCl pH 7.5, 150 mM NaCl, 0.1% Tween-20) containing 5% bovine serum albumin (BSA, Proliant Biologicals, Ankeny, IA, USA) at room temperature. All primary antibodies were diluted 1:1000 in TBST containing 5% BSA. The following primary antibodies were used: Tuberin/TSC2 (3612, Cell Signaling Tech., Danvers, MA, USA), phospho-Tuberin/TSC2 (Ser1387) (5584, Cell Signaling Tech., USA), phospho-Tuberin/TSC2 (Ser939) (3615, Cell Signaling Tech., USA), ULK1 (8054, Cell Signaling Tech., USA), phospho-ULK1 (Ser555) (5869, Cell Signaling Tech., USA), phospho-ULK1 (Ser757) (6888, Cell Signaling Tech., USA), AKT (9272, Cell Signaling Tech., USA), phospho-AKT (S473) (4060, Cell Signaling Tech., USA), Beclin-1 (3495, Cell Signaling Tech., USA), AMPK (2532, Cell Signaling Tech., USA), phospho-AMPK (T172) (2535, Cell Signaling Tech., USA), 4E-BP1 (9644, Cell Signaling Tech., USA), phospho-4E-BP1 (Thr37/46) (236B4, Cell Signaling Tech., USA), LC3 (4108, Cell Signaling Tech., USA), p62 (ab56416, Abcam, Waltham, MA, USA), GAPDH (ab8245, Abcam, USA).

Secondary HRP-conjugated anti-rabbit (1706515, Bio-Rad, Hercules, CA, USA) antibodies were used at a 1:3000 dilution. α-tubulin (1:3000, ab18251, Abcam, USA) was used as a loading control.

### 4.5. High-Performance Liquid Chromatography (HPLC)

Chemicals: acetonitrile (MeCN) (HPLC grade, Fisher Scientific), trifluoroacetic acid (TFA) (HPLC grade, Fisher Scientific), formic acid (FA) (LCMS grade, Fisher Scientific).

HPLC analyses were performed on an Agilent 1260 Infinity system equipped with a binary pump, dual-loop autosampler, fraction collector, column thermostat, and diode-array detector (DAD). Separations were carried out on a Macherey–Nagel C18 column (5 µm, 4.6 × 250 mm) maintained at 35 °C. UV detection was recorded at 210, 254, 350, and 400 nm.

Mobile phase A consisted of water with 0.1% TFA, and mobile phase B consisted of MeCN with 0.1% TFA. The injection volume was 50 µL. Elution was performed at a flow rate of 1.0 mL/min using a linear gradient from 5% to 95% B over 10 min, followed by an isocratic hold at 95% B for 4 min. The active fraction eluted at approximately 6.4 min.

### 4.6. Preparative Isolation of Salazinic Acid

Preparative purification of salazinic acid was performed on an Puriflash 4250 (Interchim, Montluçon, France) preparative chromatograph equipped with a VDSpher 100 C18-E column (250 × 20 mm, 10 µm). Aqueous 0.1% TFA and MeCN containing 0.1% TFA were used as mobile phases A and B, respectively. UV–Vis detection was carried out at 205, 254, and 550 nm. Elution was performed at a flow rate of 20 mL/min using the following program: isocratic elution with 30% MeCN for 5 min, followed by a linear gradient from 30% to 75% MeCN over 12 min, and then a linear gradient from 75% to 95% MeCN over 3 min. Salazinic acid eluted at approximately 13 min. The fraction containing salazinic acid was collected and dried under reduced pressure, yielding a beige powder. The powder was suspended in acetone using an ultrasonic bath and centrifuged at 22,000× *g*; the supernatant was discarded. This washing procedure was repeated three times. The precipitate was thoroughly dried under reduced pressure to afford salazinic acid as an off-white powder. 

The ^1^H and ^13^C NMR spectra of the pure compounds were recorded on a Avance-700 MHz (Bruker Corporation, MA, USA) spectrometer using TMS as an internal standard. The chemical shifts were expressed as δ values in parts per million (ppm), and the coupling constants (*J*) were given in Hertz (Hz).

5,13,17-trihydroxy-12-(hydroxymethyl)-7-methyl-9,15-dioxo-2,10,16-trioxatetracyclo[9.7.0.03,8.014,18]octadeca-1(11),3(8),4,6,12,14(18)-hexaene-4-carbaldehyde (Salazinic acid) 1H NMR (700 MHz, DMSO-*d*_6_) δ 12.04 (br s, 1H), 10.45 (s, 1H), 10.22 (br s, 1H), 8.28 (br s, 1H), 6.87 (s, 1H), 6.81 (br s, 1H), 5.32 (br s, 1H), 4.65 (s, 2H), 2.45 (d, J = 0.8 Hz, 3H). ^13^C NMR (DMSO-*d*_6_, 176 MHz) δ 192.8, 165.9, 164.0, 163.6, 160.3, 152.2, 138.0, 137.3, 123.4, 117.4, 111.9, 110.7, 109.7, 52.7, 21.4. Pictures of NMRs with additional experiments (COSY and HSQC-^13^C) are available in the Supporting Information (Figure S5), as well as a comparison between chemical shifts and literature spectra (Tables S1 and S2). 

Purity was confirmed by analytical HPLC (Figure S6).

### 4.7. High-Resolution Liquid Chromatography-Mass Spectrometry (HR-LCMS)

HR-LCMS analysis. The analysis was performed on an UltiMate 3000 Acclaim RSLC HPLC system (Thermo Fisher Scientific, Waltham, MA, USA) with a 120 C18 2.2 um 2.1 × 100 mm column, gradient of acetonitrile (0.1% FA) in water (0.1% TFA) 5–95% for 10 min, connected to a Bruker maXis II 4G ETD (Bruker, Billerica, MA, USA). Spectra recording mode: ESI ionization mode, full scan from 100 to 1500 *m/z*, MS/MS with selection of the three most intense ions, dissociation type: CID 10–40 eV, collision gas: nitrogen. The obtained mass spectra were analyzed using mzMine 4.8.40 software [56] and ToppView [57]. All graphical representations were further refined and finalized in Adobe Illustrator 2024.

### 4.8. Transmission Electron Microscopy

Mouse neuroblastoma (N2a) cells were cultured on glass slides in a standard growth medium. The cells were treated with salazinic acid at a concentration of 100 µg/mL or with 0.1% DMSO as a control. After 24 h of incubation, the culture medium was removed, and the cell-containing slides were fixed in 3% glutaraldehyde (Sigma-Aldrich, St. Louis, MO, USA) prepared in phosphate buffer (pH 7.4) for 2 h at 4 °C. This was followed by post-fixation in 1% osmium tetroxide in phosphate buffer (pH 7.4) for 1.5 h.

Subsequently, the cells were dehydrated through a graded series of alcohol solutions, with the 70% alcohol step saturated with uranyl acetate (Serva, Heidelberg, Germany) to enhance sample contrast. The specimens were then embedded in Epon-812 epoxy resin. Serial ultrathin sections were prepared using a diamond knife on a ULTRACUT UCT ultramicrotome (Leica, Wetzlar, Germany). Imaging was performed using a JEM-1400 electron microscope (Jeol, Tokyo, Japan).

For the statistical analysis of autophagy rate, a previously published procedure was used [58]. Briefly, random electron micrographs of at least 17 cells treated with salazinic acid or DMSO were selected. On each image, pixels corresponding to autophagosomes and to the entire cell area were manually delineated at the pixel level. The delineated regions were then analyzed using Adobe Photoshop 2025 software. The areas of autophagosomes and the total cell area were quantified in pixels using the measurement tool. For each image, the fraction of the total cell area occupied by autophagosomes was then calculated.

### 4.9. Cytotoxicity Assay

Human neuroblastoma SH-SY5Y and embryonic human kidney HEK293 cells were seeded at a density of 20,000 cells per 100 μL DMEM/F12 medium per well in 96-well plates. The maximum concentration used for salazinic acid was 500 μg/mL, and the final DMSO concentration did not exceed 0.5% (*v*/*v*).

Cell viability was assessed using the resazurin assay after 24 h of compound treatment. Cells were incubated with resazurin (0.15 mg/mL, Thermo Fisher Scientific, USA) for 2 h at 37 °C in a CO_2_ incubator. After incubation, fluorescence was recorded using a 560 nm excitation/590 nm emission filter set with a VICTOR X5 plate reader (PerkinElmer, Waltham, MA, USA).

Cytotoxicity data were analyzed using nonlinear regression in GraphPad Prism 9, applying a four-parameter logistic model (lower limit, upper limit, slope, and CC_50_).

## Data Availability

The original contributions presented in this study are included in the article/Supplementary Material. Further inquiries can be directed to the corresponding authors.

## Supplementary Materials

The following supporting information can be downloaded at: https://www.mdpi.com/article/10.3390/ijms27198621/s1.
