# Supplementary material for: The Reindeer’s Secret: Salazinic Acid from Lichens as a Candidate Modulator of Autophagy
Source: Int J Mol Sci. 2026 Sep 26;27(19):8621. doi: 10.3390/ijms27198621 (PMC13665029; doi:10.3390/ijms27198621)
Supplement: Supplementary file 1 [file ijms-27-08621-s001.zip › ijms-4504853-supplementary.pdf]

**Supplementary Materials for the Article: The Reindeer's Secret: Salazinic Acid from Lichens as a Candidate Modulator of Autophagy**

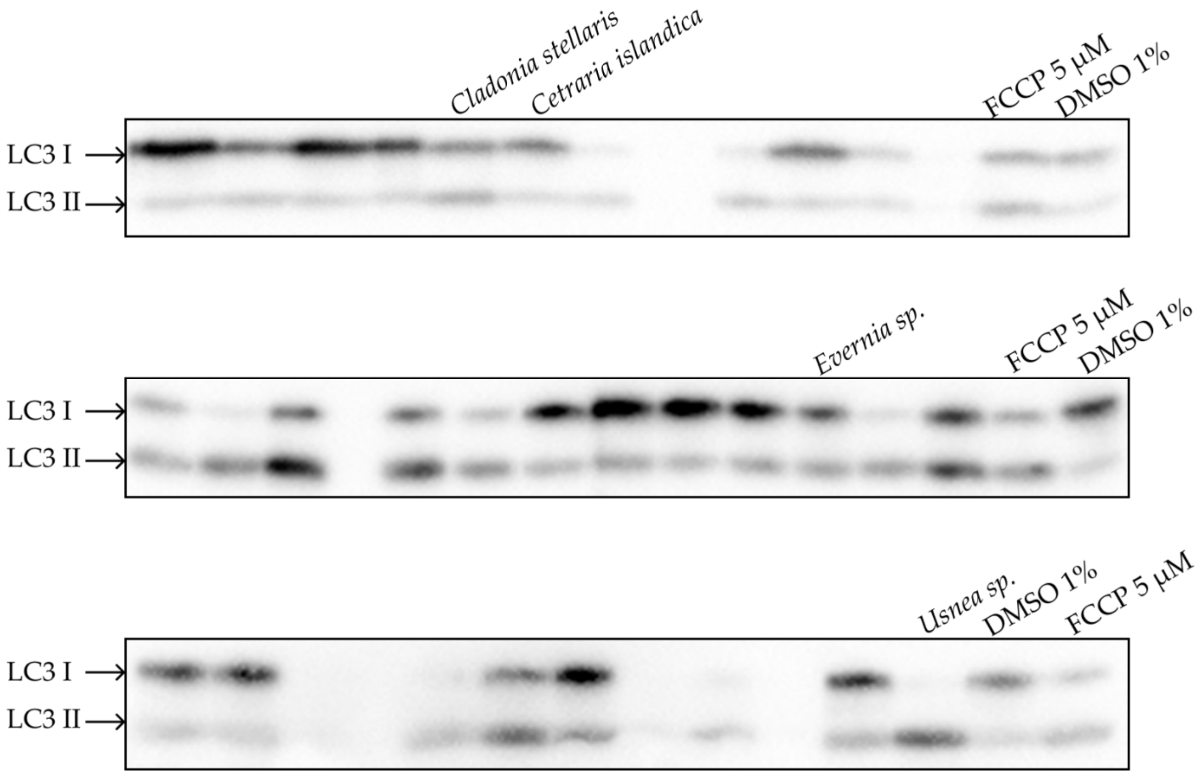


**Figure S1**. Analysis of the ability of ethanolic extracts of various plants to induce autophagy in SH-SY5Y cells; analysis was performed by immunoblotting with LC3-I/II staining. FCCP at 5 µM was applied to cells as a positive control, and medium with 1% DMSO was used as a negative control.

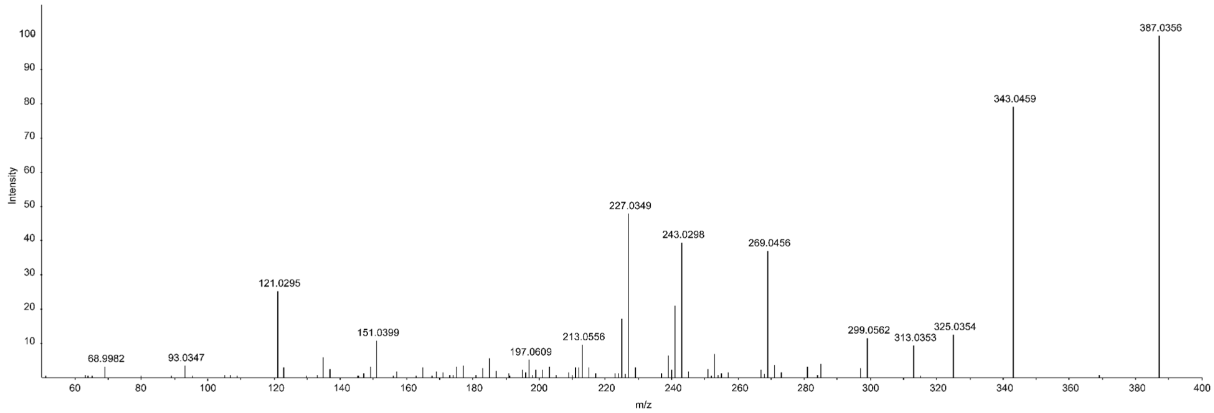


**Figure S2**. MS/MS fragmentation spectra for the compound isolated from *Usnea sp.* ethanolic extract. Negative-ion mode, CID spectrum of the precursor ion at m/z 387.0353.

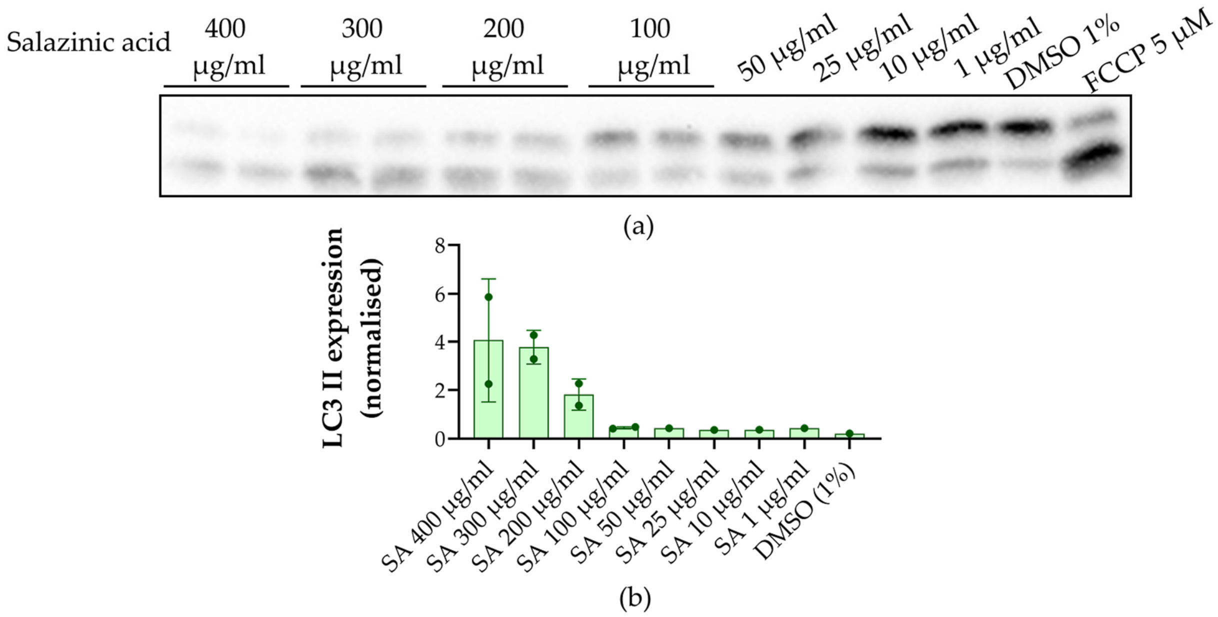


**Figure S3**. Analysis of the activity of salazinic acid to induce autophagy in SH-SY5Y cells (wide concentration range). (a) Immunoblotting analysis of autophagy activity in cells after treatment with salazinic acid at different concentrations or DMSO 1% for 16 hours. (b) Bar graph indicating the LC3 II/I ratio in cells after treatment with salazinic acid at different concentrations normalized to the corresponding ratio in negative controls (DMSO 1%). The ratio was used as a comparative screening parameter for extracts tested in parallel and was not used as a measure of autophagic flux. Here and further: each dot on the blot represents an independent biological replicate. SA - salazinic acid.

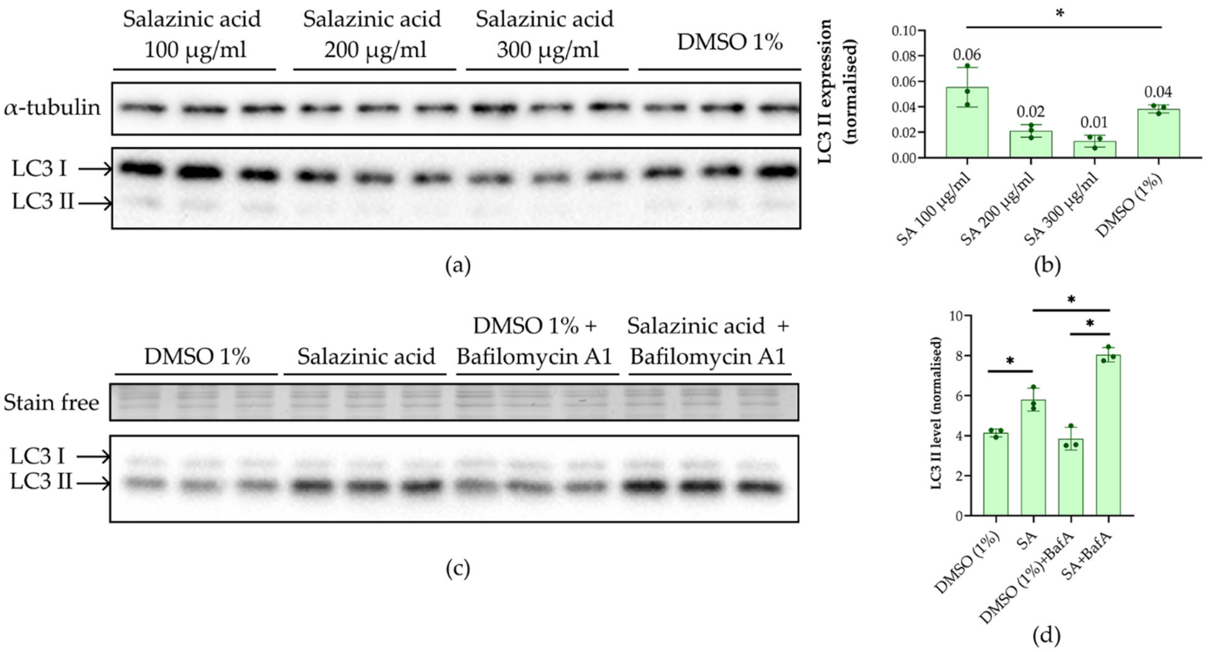


**Figure S4**. Analysis of the activity of salazinic acid to induce autophagy in SH-SY5Y and N2a cells. (a) Immunoblotting analysis of autophagy activity in SH-SY5Y cells after treatment with salazinic acid at different concentrations or 1% DMSO for 8 hours. (b) Bar graphs indicating levels of LC3 II normalized to α-tubulin in cells after treatment with salazinic acid at different concentrations or 1% DMSO for 8 hours. (c) Immunoblotting analysis of autophagic flux in N2a cells after treatment with salazinic acid (100 µg/ml) or DMSO 1% for 16 hours in the presence or absence of Bafilomycin A1 (2 hours, Baf. A1). (d) Bar graph indicating levels of LC3 II normalized to stain-free total protein in cells after treatment with salazinic acid (100 µg/ml) or DMSO 1% for 16 hours in the presence or absence of Bafilomycin A1 (2 hours, BafA). The results of a one-sided Mann-Whitney U test are indicated on the graph: * represents p-value = 0.05. SA-salicylic acid.

**Figure S5**. NMR spectra

Molecule numbering for NMR spectra

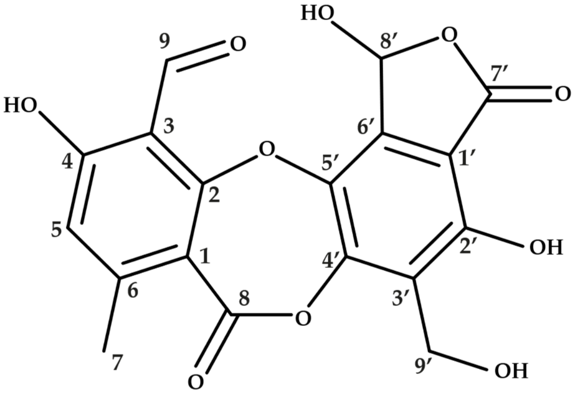


$^1H$, 700 MHz, DMSO-$d_6$

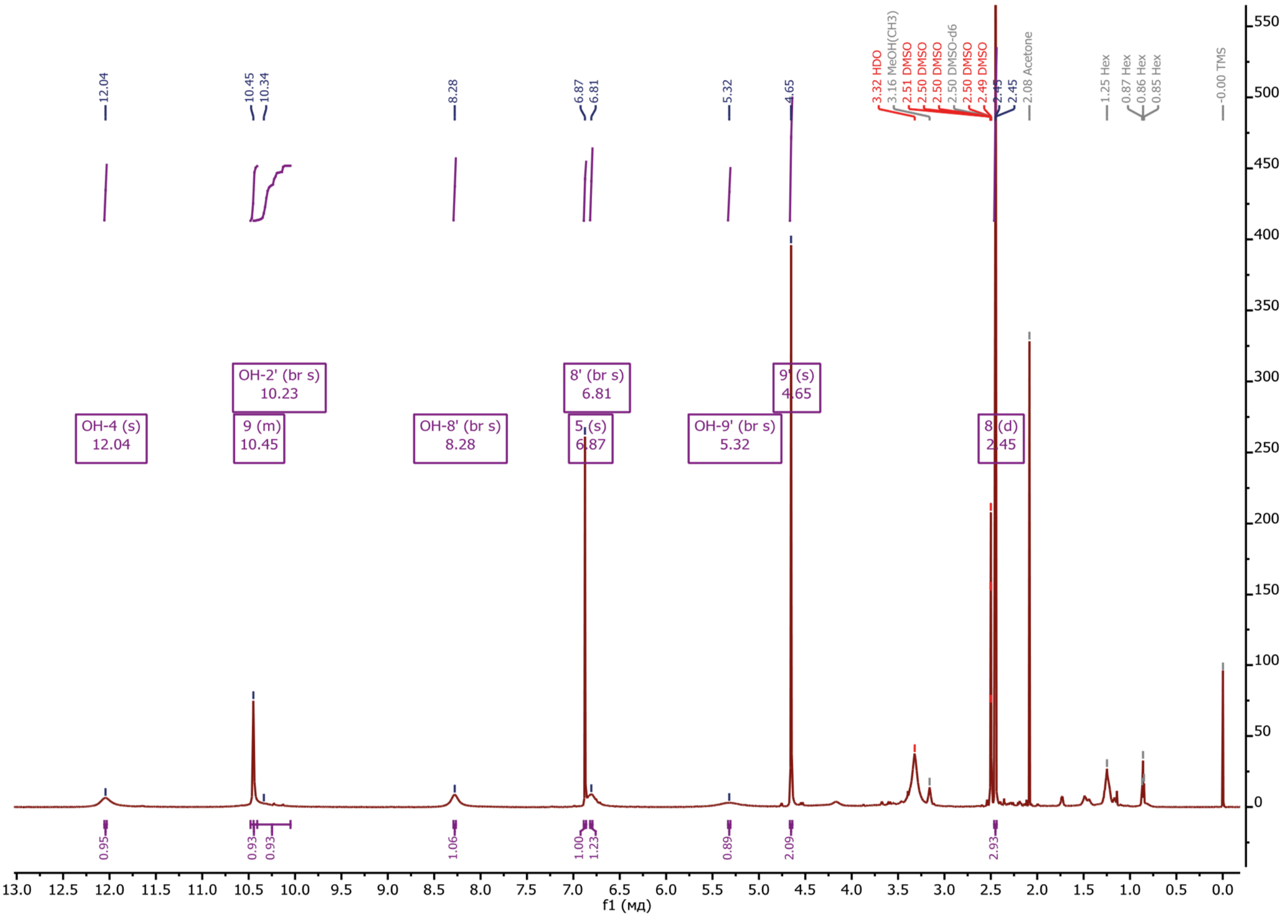

$^{13}$C, 176 MHz, DMSO-$d_6$

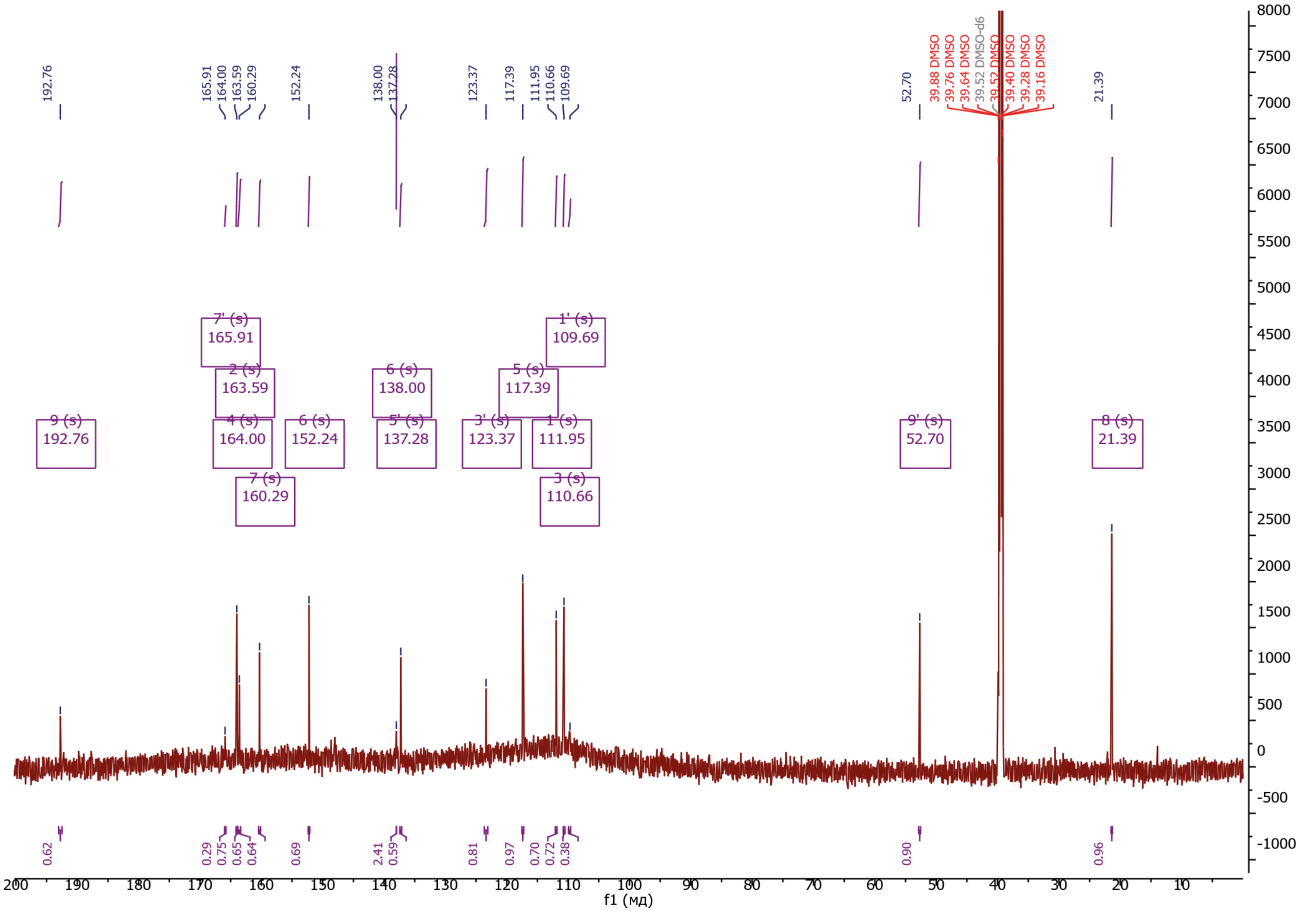

COSY, DMSO-$d_6$

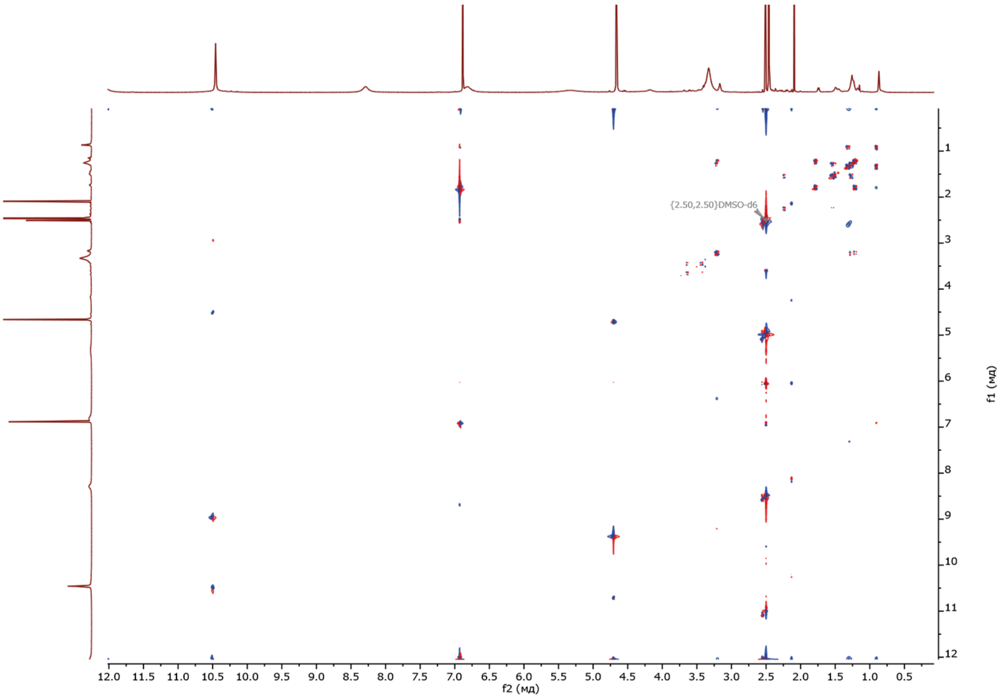
{2.50,2.50}DMSO-d6
12.0 11.5 11.0 10.5 10.0 9.5 9.0 8.5 8.0 7.5 7.0 6.5 6.0 5.5 5.0 4.5 4.0 3.5 3.0 2.5 2.0 1.5 1.0 0.5
f2 (мд)
1 2 3 4 5 6 7 8 9 10 11 12
f1 (мд)

HSQC-$^{13}$C, DMSO-$d_6$

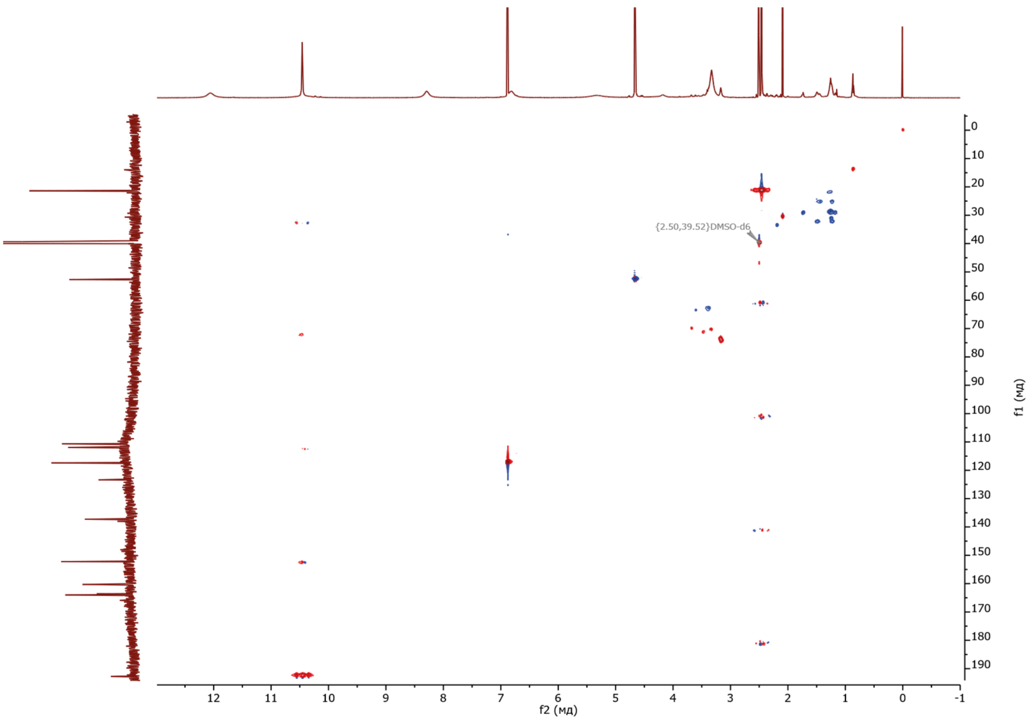


**Figure S6.** HPLC analysis of the isolated compound.

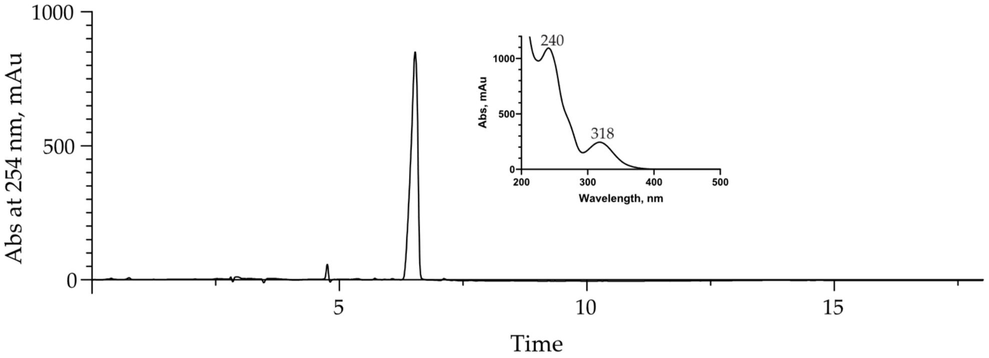


Column – C18 Mancherney Nagel 4.6 x 250, 5 µm. Gradient elution from 5% MeCN in water to 95% MeCN over 10 min, then 8 min at 95% MeCN. Injection volume: 25 uL. Detection at 254 nm. Resulted purity (by % of total absorption) – 98%.

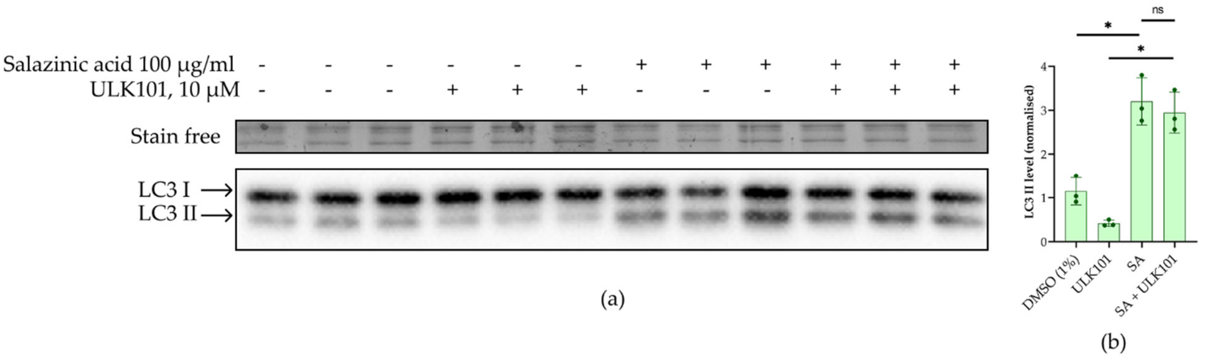


**Figure S7.** Salazinic acid retains its ability to increase LC3-II levels under conditions of combined AMPK and ULK1 inhibition. (a) Immunoblotting analysis of the autophagy marker LC3 in AMPK-knockout SH-SY5Y cells treated with salazinic acid in the presence or absence of the ULK1 inhibitor ULK101 (10 μM). (b) Bar graph indicating levels of LC3 II normalized to stain-free total protein. The results of a one-sided Mann-Whitney U test are indicated on the graph: * – p-value = 0.05. SA - salazinic acid

**Table S1**. $^{1}$H NMR Shifts table against literature reference [38]. Measured in DMSO-$d_6$, literature in $CDCl_3$- DMSO-$d_6$ (1:3)

| | δ, $^{1}$H, measured, ppm | $^{1}$H multiplicity, intensity, J measured | δ, $^{1}$H, literature, ppm | $^{1}$H multiplicity, intensity, J literature |
|---|---|---|---|---|
| OH-4 | 12,04 | br s, 1H | 12,12 | s |
| 5 | 6,87 | s, 1H | 6,84 | m, 1H |
| 8 | 2,45 | d, 3H, **J = 0.8** | 2,47 | d, 3H, **J = 0.8** |
| 9 | 10,45 | s, 1H, m | 10,5 | d, 1H, **J = 0.5** |
| OH-2' | 10,23 | br s | 10,38 | br s |
| 8' | 6,81 | br s | 6,78 | br s,1H |
| OH-8' | 8,28 | br s, 1H | 8,3 | br s |
| 9' | 4,65 | s, 2H | 4,7 | s, 2H |
| OH-9' | 5,32 | br s, 1H | | |

**Table S2**. $^{13}$C NMR Shifts table against literature reference [38]. Measured in DMSO-$d_6$, literature in $CDCl_3$- DMSO-$d_6$ (1:3)

| | δ, $^{13}C$, measured, ppm | δ, $^{13}C$ ,literature, ppm |
|---|---|---|
| 1 | 112 | 111,9 |
| 2 | 163,6 | 163,9 |
| 3 | 110,7 | 110,3 |
| 4 | 164 | 164,9 |
| 5 | 117,4 | 117,5 |
| 6 | | 152,8 |
| 7 | 160,3 | 160 |
| 8 | 21,4 | 21,3 |
| 9 | 192,8 | 193,5 |
| 1' | 109,7 | 109,6 |
| 2' | | 153,3 |
| 3' | 123,4 | 122,5 |
| 4' | | 147,9 |
| 5' | 137,3 | 137,66 |
| 6' | 138 | 137,5 |
| 7' | 165,9 | 166,3 |
| 8' | | 95,2 |
| 9' | 52,7 | 54,2 |
